# A dynamic network of transcription in LPS-treated human subjects

**DOI:** 10.1186/1752-0509-3-78

**Published:** 2009-07-28

**Authors:** Junhee Seok, Wenzhong Xiao, Lyle L Moldawer, Ronald W Davis, Markus W Covert

**Affiliations:** 1 Stanford Genome Technology Center, Palo Alto, California, USA; 2 Department of Electrical Engineering, University, Stanford, California, USA; 3 Department of Bioengineering, Stanford University, Stanford, California, USA; 4 Department of Surgery, University of Florida, Gainesville, Florida, USA

## Abstract

**Background:**

Understanding the transcriptional regulatory networks that map out the coordinated dynamic responses of signaling proteins, transcription factors and target genes over time would represent a significant advance in the application of genome wide expression analysis. The primary challenge is monitoring transcription factor activities over time, which is not yet available at the large scale. Instead, there have been several developments to estimate activities computationally. For example, Network Component Analysis (NCA) is an approach that can predict transcription factor activities over time as well as the relative regulatory influence of factors on each target gene.

**Results:**

In this study, we analyzed a gene expression data set in blood leukocytes from human subjects administered with lipopolysaccharide (LPS), a prototypical inflammatory challenge, in the context of a reconstructed regulatory network including 10 transcription factors, 99 target genes and 149 regulatory interactions. We found that the computationally estimated activities were well correlated to their coordinated action. Furthermore, we found that clustering the genes in the context of regulatory influences greatly facilitated interpretation of the expression data, as clusters of gene expression corresponded to the activity of specific factors or more interestingly, factor combinations which suggest coordinated regulation of gene expression. The resulting clusters were therefore more biologically meaningful, and also led to identification of additional genes under the same regulation.

**Conclusion:**

Using NCA, we were able to build a network that accounted for between 8–11% genes in the known transcriptional response to LPS in humans. The dynamic network illustrated changes of transcription factor activities and gene expressions as well as interactions of signaling proteins, transcription factors and target genes.

## Background

An achievement that would have a major impact on our understanding of transcriptional regulatory networks would be to map out the coordinated dynamic responses of signaling proteins, transcription factors and target genes over time. The primary challenges to such an effort are development of high-throughput technologies to measure transcription factor activities at the genome-scale, and computational tools to interpret the data and predict the structure and dynamics of the underlying networks.

Recent development of high-throughput technologies has enabled large-scale measurements of biological signals related to transcription, such as the expression of target genes and the activities of transcription factors. For target gene expression, microarrays measure the expression levels of thousands of genes simultaneously [1-3]. However, efforts to broadly assess transcription factor activities on a genome wide scale are much more limited. Technologies such as chromatin immunoprecipitation-on-a-chip can identify all of the DNA binding sites occupied by a single transcription factor for a given condition [4,5]. Flow cytometry can also be used to determine transcription factor activities by labeling active factors with fluorescently labeled antibodies [6], but throughput is limited by the number of available antibodies and colors. As yet, there is no transcription factor-focused equivalent of the gene expression array, which would enable monitoring of all transcription factor activities at a time. Such technology would be critical to generating a complete dynamic network of transcription empirically.

To compensate for this inability to assay transcription factor activity at the large scale, there have been several efforts to infer regulatory networks computationally [7]. One of these approaches, called Network Component Analysis (NCA), is a method for determining both activities and regulatory influence for a set of transcription factors with known target genes [8]. NCA has been successfully applied in several areas. It was used to identify previously unnoticed oscillatory activity patterns in the yeast cell cycle [8], as well as to generate a predicted activation time course of catabolite repressor protein in *Escherichia coli*, which was verified experimentally [9]. More recently, NCA was used to predict activities of important transcription factors like sterol regulatory element-binding proteins and peroxisome proliferative-activated receptors in a mouse knockout model of human glycerol kinase deficiency [10,11]. In parallel, several studies have expanded and strengthened NCA as a computational tool [12-14].

In eukaryotic systems, inflammation and activation of innate immunity are fundamental host responses to microbial invasion and endogenous danger signals. Blood leukocytes contribute to this inflammatory response, and exposure to a prototypical stimulus such as LPS leads first to changes in gene expression, then production of cytokines which are secreted and cause secondary transcriptional and other responses [15]. In previous work, we and others generated a set of gene expression profiles from human subjects over 24 hours following the intravenous administration of bacterial endotoxin LPS [16]. Experimental endotoxicosis produces in the previously healthy individual a transient but significant systemic inflammatory response, characterized by fever, tachycardia, malaise, and a hepatic acute phase response. Administration of endotoxin is presumed to model the early inflammatory changes associated with a microbial invasion, sepsis and the systemic inflammatory response syndrome [17]. We used this data to determine important clusters of genes involved in the early inflammatory response, as well as to depict the temporal changes in gene expression as inflammation resolved over the first twenty-four hours. In this study, we calculated transcription factor activities and regulatory influences in the above dataset using NCA, and interpreted the results to develop a dynamic network of transcription events following experimental endotoxicosis in humans.

## Results and Discussion

**Figure 1:**
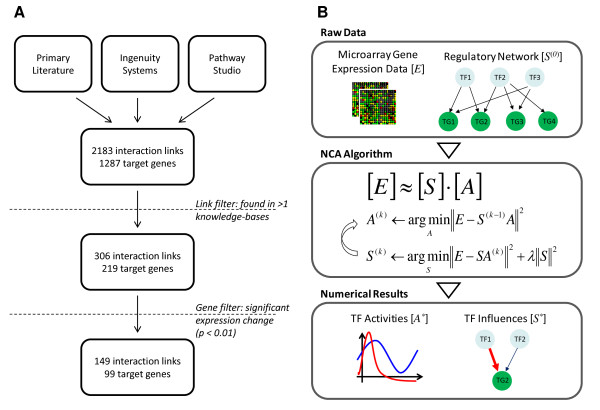
**Schematic of the approach**. (A) Flowchart describing the steps to reconstruct our initial transcriptional regulatory network. (B) A set of gene expression profiles (matrix *E*) and about a proposed structure for the underlying transcriptional regulatory network (matrix *S*^(0)^) are used as inputs for Network Component Analysis (NCA). NCA uses an algorithm that first calculates the expected transcription factor activities (matrix *A*), and then recalculates *S *based on the new values of *A*, until both matrices converge. The outputs of this procedure are A* and S*, final values of *A *and *S*, which provide information about transcription factor activity as well as regulatory structure, respectively.

Our approach follows the schematic in Figure 1. NCA requires two inputs: a set of gene expression profiles and a pre-defined regulatory network, which is a matrix that contains initial estimates of the influence each transcription factor on the target genes. The original gene expression data set is obtained from Calvano *et al *[16], in which peripheral blood leukocytes were obtained from four different individuals prior to and at five time points after injection with endotoxin, 24 profiles in total.

To define a regulatory network which could account for a significant percentage of the gene expression response, we identified a set of key transcription factors previously known to be involved in the LPS response, together with a set of known target genes for these factors. Ten transcription factors were chosen for our study (listed here by gene name for continuity). NFKB1 (encoding p50/p105), RELA (encoding p65) and IRF3 were chosen as factors involved in the primary response to endotoxin. Endotoxin binding to Toll-like receptors (TLR) leads to activation of NF-κB dimers, among which p65:p50 is common [18]. LPS stimulation also induces IRF3 activation through TLR4 [19]. These transcription factors induce expression of several cytokines which can further activate a secondary transcription response through factors such as STAT1, 3 and 6 [15,20]. CREB1 is activated by LPS through the p38 kinase-SAPK2 pathway [21]. It is known that LPS activates AP-1 complexes consisting of FOS, JUN, JUNB and JUND [22]. Among these factors, JUN and FOS were chosen for our model. The role of MYC in inflammation response is poorly understood [23]; however, many genes connected to MYC showed significant changes in their expression levels in the original study and so we included it as well [16].

To identify established regulatory interactions between these transcription factors and target genes, we relied largely on the primary literature [15,16,20,22,24-28] (Figure 1A). However, two knowledge-bases were also used: Ingenuity Systems  and Pathway Studio [29]. Both the Ingenuity and Pathway Studio knowledge-bases consist of regulatory relationships parsed from MEDLINE abstracts; the Ingenuity knowledge-base also includes information from manually-curated peer-reviewed publications. For our ten transcription factors, this strategy resulted in a list of 1,287 target genes, with 2,183 interactions between transcription factors and target genes. To reconcile differences in these different sources of regulatory network information (literature, Ingenuity, Pathway Studio), we only included an interaction in our network if it could be identified in two out of the three resources. This filtering process reduced our list to 219 target genes regulated by 306 interactions with the ten transcription factors. To focus on the most useful expression information, we only considered target genes for which expression changed significantly over time (p-value < 0.01).

The network for the inflammatory response finally included 10 transcription factors, 99 target genes and 149 regulatory relations. This network can be represented in matrix form, with a density of ~15%, or 149 relations/(10 factors × 99 targets). In contrast, the expected density of a genome-wide regulatory relationship matrix, given our current state of knowledge about human transcriptional regulation would be about 0.1% (~20,000 relations in Ingenuity Systems and Pathway Studio databases, ~1,000 transcription factors and ~20,000 target genes). Our network density is therefore relatively high, reflecting the comparatively high level of research interest in this system.

We estimated the activation of the transcription factors in our network over time using NCA (Figure 1B). NCA decomposes a matrix containing gene expression values (*E*) into a matrix which represents the influence of a transcription factor on a target gene (strength matrix *S*) and a matrix which contains the transcription factor activities (activity matrix *A*) [8]. We found that both outputs of NCA – predicted factor activities *A *and regulatory influences *S *– have added additional insights to gene expression data where the underlying regulatory network structure is partially known.

### Transcription factor activities

**Figure 2:**
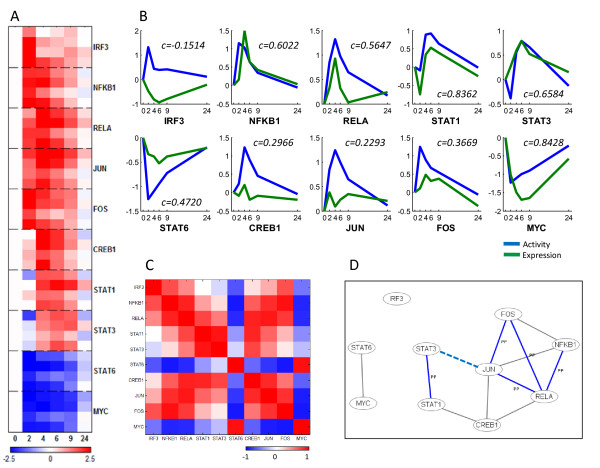
**Transcription factor activities calculated using NCA**. (A) Predicted activities of the ten transcription factors used in this study. For each transcription factor, rows represent progression in time and columns correspond to the four human subjects. Activities of each row are normalized to the zero time point. (B) Transcription factor activities (blue) compared to gene expression (green), with Pearson correlation coefficients noted. Both activity and expression at each time point are averages normalized to the time = 0 values, and the activity is further scaled for direct comparison with the expression values. (C) Correlation matrix between transcription factor activities. Red represents positive correlation, and blue represents negative correlation. (D) Inferred combinatorial regulation pairs of transcription factors. A blue solid line indicates that the pair was supported by protein-protein interaction knowledge of BIND and high correlation of their activities (>0.75). A black solid line indicates that the pair was only supported by high correlation, and a blue dotted line indicates that the pair was only supported by the interaction database.

Figure 2A and 2B show the estimated activities of our 10 transcription factors. Transcription factor activities clearly showed early-, mid-, and late-phase action in response to LPS. IRF3, NFKB1(p50/p105) and RELA(p60) were activated within 2 hours after the endotoxin was injected. IRF3 activation peaked at 2 hours and returned to its base level at 4 hours. NFKB1 and RELA were also activated early but decreased in activity more slowly. These three factors can induce expression of tumor necrosis factor alpha, which then further activates the NF-κB complex [25,26], and could contribute to the extended NF-κB activation. JUN and FOS are known to be activated through the JNK pathway [30,31], and had a similar activation profile to NFKB1 and RELA. In contrast, STAT1, STAT3 and CREB1 exhibited a late-phase response. The STAT1 and STAT3 predictions correspond to previous findings that STATs are activated by cytokines transcribed by the NF-κB complex [15]. It was surprising that predicted CREB1 activation peaked at four hours, given that previous reports detect phosphorylated CREB at 30 minutes [32]. However, the prediction was the result of late-phase induction of known CREB-dependent gene expression, such as ALAS1 and CEBPD [33,34]. Both STAT6 and MYC were predicted to be somewhat deactivated over nine hours. Deactivation of STAT6 was predicted due to repression of MHC-II class genes which are known to be regulated by STAT6 [35], as well as the expression of SOCS1, which has been reported to lead to deactivation of STAT6 [36]. MYC expression can be decreased through a STAT1-dependent pathway under IFN-γ stimulation conditions [37], and it is possible that the deactivation predicted here depends on STAT1 as well.

Transcription factor activities are sometimes, but not always, correlated with the gene expression of the factor. We compared the calculated transcription factor activities with the gene expression data for each factor (Figure 2B). NFKB1, RELA, STAT1, STAT3 and MYC showed strong positive correlation between activities and expression (correlation coefficient *c *> 0.56), possibly due to auto- or cross-regulation. For example, NFKB1 activity and expression are tightly correlated (*c *= 0.6022), possibly because the NF-κB p65:p50 complex can regulate NFKB1 [38,39]. STAT1 activity and expression are also strongly correlated (*c *= 0.8362), which might relate to the transcriptional effect of STAT3 on STAT1 expression [40], particularly given that STAT1 and STAT3 have highly correlated activities (Figure 2C, *c *= 0.9329). On the other hand, the activities and expression show lower or no correlation for IRF3, JUN, FOS and CREB (-0.15 <*c *< 0.37).

The linear model of gene expression upon which NCA rests does not account for the interactions between transcription factors. However, we wondered if the NCA-predicted correlation in transcription factor activities could be due to the combined action of two transcription factors, either as a complex or otherwise. We therefore checked transcription factor pairs with significant activity correlation to published protein-protein interactions catalogued in the Biomolecular Interaction Network Database (BIND) [41]. Interestingly, transcription factors known to act together showed high correlation in their activity profiles (Figure 2D). For example, highly correlated transcription factors NFKB1(p50/p105) and RELA(p65) regulate their target genes as a p65:50 heterodimer form [42], and STAT1 and STAT3 are also known to act as a dimer [20], as are JUN and FOS [30]. Additionally, some transcription factors (STAT1 and CREB1, STAT6 and MYC) showed a positive correlation in their activity even though they are not known to form a complex with other transcription factors. Transcription factors can have similar – and even coordinated – activities without direct interaction, so it may be that these latter predictions reflect an indirect interaction.

On the other hand, it is possible that some of the correlated transcription factor activities may be based on incorrect NCA predictions. The largest possible source of error for NCA decomposition is the initial connectivity matrix, which is based on the current, generally incomplete or erroneous, understanding of the human transcriptional regulatory network. The effect of missing or false data in the connectivity matrix is hard to predict in advance. However, the sensitivity of NCA to the connectivity matrix can be estimated by adding or removing connections randomly from the original matrix, and repeating the NCA calculation multiple times [14]. Using this approach, we found that transcription factor activities predicted by NCA and our original connectivity matrix were robust, even if 10–15% of the connectivity matrix contained inaccurate connections (Table 1). Given that our matrix was limited to only high-confidence interactions, this level of sensitivity was assumed to be tolerable.

**Table 1:** NCA simulation with random noisy connections

	1%	5%	10%	15%	20%
Mean(Corr)	0.9892	0.9548	0.9330	0.9107	0.8880
SD(Corr)	0.0678	0.1258	0.1378	0.1579	0.1662

NCA simulation was performed based on the original network model with 1~20% of random noisy connections. A pair of a transcription factor and a target gene was randomly selected; a random connection was added if the original connection did not exist, or removed otherwise. For each percentage of random connections, the simulation was repeated by 100 times. Mean and standard deviation of activity correlations with the original noise-free network model were calculated.

### Regulatory influence matrix and gene expression clustering

**Figure 3:**
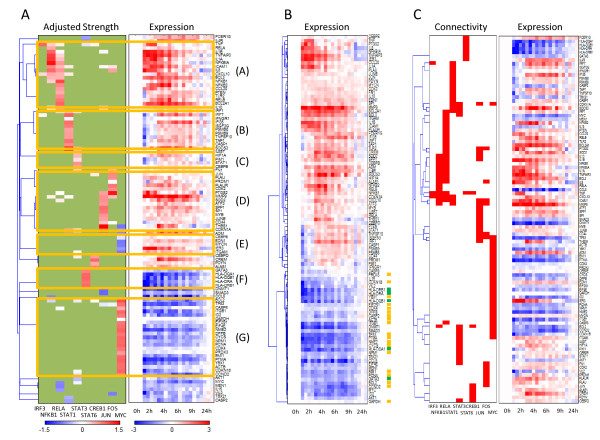
**Hierarchical clustering in the context of a defined regulatory network**. (A) The adjusted strength matrix was used for clustering, after which the gene expression matrix was appended. Seven major clusters which have more than five associated genes are highlighted. In the adjusted strength matrix heatmap, green color indicates that there is no prior regulatory connection in our model while white color indicates a weak regulatory influence. (B) Clustering with gene expression only. Genes in the Cluster F(regulated by STAT6) were noted with green dots, and genes in the Cluster G(regulated by MYC) were noted with orange dots. (C) Clustering with the binary regulatory relations (initial connectivity matrix) assuming all regulatory strengths are equal.

We thought that the adjusted strength matrix might be used to enhance typical gene expression clustering techniques. Signed quantitative values of the adjusted strengths were able to form more biological meaningful clusters beyond the prior binary regulatory connections. In Figure 3A, target genes were hierarchically clustered with the adjusted strengths of transcription factors and shown with gene expression. We identified seven major clusters, which correlate to the coordinated action of transcription factors to regulate gene expression. Cluster A highlights the influence of NFKB1(p50/p105) and RELA(p65) on a set of eighteen genes. Interestingly, some genes are linked to p65 only, suggesting that these genes may be under the specific control of the p65:p65 homodimer, rather than the p65:p50 heterodimer. For example, the cluster suggests that CXCL10 expression depends on both p65 and p50, which has been demonstrated experimentally in NFKB1^-/- ^and RELA^-/- ^knockout mice [43]. Clusters B and C contain the genes regulated by STATs 1 and 3, while Cluster D genes are regulated by JUN and FOS. Clusters E and G are primarily regulated by MYC, but with repression in E and induction in G. Cluster F genes are regulated by STAT6. All of the transcription factors known to act in dimers [20,30,44] – the NF-κB complex of NFKB1-RELA, as well as STAT1-STAT3 and JUN-FOS – were either in the same cluster or closely adjoining clusters, and had correlated activation profiles. However, although STAT6 and MYC had correlating activation profiles, the genes under their influence (Clusters E, F and G) did not cluster closely. Therefore, when studied together, activation profiles and regulatory influences may provide insight on the coordination between transcription factors.

Although our clustering was based on the matrix of regulatory influence, the clusters also provided a strong basis for interpreting gene expression. Pair-wise correlation tests on expression between genes within a cluster showed significantly higher average correlation than random clusters (Table 2). Furthermore, the resulting gene expression clusters can be immediately linked to the specific transcription factors whose action created the expression profile. Importantly, clustering by transcription strength can identify new clusters unobtainable by clustering the expression data alone. For example, Cluster F and G could not be distinguished when the same clustering method was applied to the gene expression data alone (Figure 3B). However, they formed unmistakable clusters from the regulatory strength matrix, being linked to the regulatory influence of either STAT6 or MYC. Furthermore, our clusters required the NCA-processed strength matrix, and could not be obtained from the initial connectivity matrix, the clustering of which led to groups of genes that did not show common expression patterns (Figure 3C). We conclude that the estimated transcription factor regulatory strengths can provide unique insights with regard to the regulation underlying gene expression, even between genes with similar expression.

**Table 2:** Major clusters formed from the adjusted strength matrix.

Cluster	Num. of genes	Dominant transcription factors	Avg. of pair-wise correlations	P-value
A	18	NFKB1, RELA	0.5742	<0.001*
B	11	STAT1	0.7815	<0.001*
C	5	STAT3	0.5138	0.025
D	17	JUN, FOS	0.4054	0.001
E	6	MYC	0.4350	0.031
F	5	STAT6	0.7546	0.004
G	21	MYC	0.6442	<0.001*

For *N *genes in a cluster, *N*(*N*-1)/2 pair-wise Pearson's correlations on expression were measured and their average was calculated. Next, *N *genes were randomly selected from a set of ~18,000 human genes and the average pair-wise correlation was calculated from the random gene set. The distribution of the average pair-wise correlation of random genes was re-estimated 1,000 times to generate a null distribution of the average pair-wise correlations. A p-value for each cluster was estimated by counting the number of random gene sets for which the average correlation is larger than the cluster correlation. *No random gene set that exceeded the cluster was found in 1,000 repeats.

### Correlation test and prediction over extended regulatory sets

**Figure 4:**
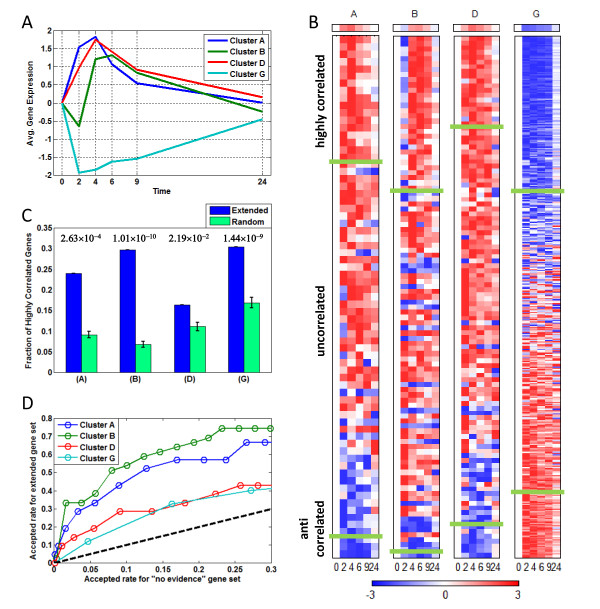
**Identification of new target genes for major clusters**. (A) The average expression profiles of the four clusters with > 10 members. (B) Expressions of extended regulatory genes sorted by correlation coefficients(*c*) with the average expression profile of a cluster. Each extended gene group was divided into highly correlated (*c *> 0.5), un-correlated (t0.5 <*c *< 0.5) and anti-correlated (*c *< t0.5) groups. The average gene expression of each cluster is shown as a row at the top of each column. (C) Ratio of highly correlated genes (*c *> 0.5) in the sets of extended regulatory genes and 1,000 randomly chosen genes. Error bars were calculated as the standard deviation of a population derived from 100 repeated tests. P-values measured by the Fisher's exact test are noted above each column set. (D) Fraction of acceptable new predicted cluster genes from both the extended and "no evidence" gene sets. Significantly expressed genes (*p *< 0.01) in both sets were plotted against each other using a range of Pearson's coefficient cutoff values for Clusters A, B, D, and G. The dashed line indicates where the fraction of acceptable genes is equal from both the extended and "no evidence" sets

The clusters shown in Figure 3A suggested that we might be able to use our cluster information to discover new regulatory relationships. We first determined the average normalized expression pattern of the genes in each cluster (= model gene group). The expression vector for each gene was normalized to have zero mean and a standard deviation of 1, and then normalized gene expression sets were averaged for each cluster (Figure 4A). We then divided all human genes measured on the expression array into three groups: those for which we had high-confidence regulatory information linking the dominant transcription factors in the cluster to the gene (model genes); genes for which we had lower-confidence regulatory information (found in only one of the two knowledge-bases), but could still be valid to extend our model (extended genes); and genes where we found no evidence of regulation by the cluster transcription factors (no-evidence genes). If a cluster had more than two dominant transcription factors, only genes which had established regulatory interactions with all factors were collected for the extended gene group.

We first wanted to see if a gene in the extended gene group had similar expression to a cluster (Figure 4B). First, Pearson's correlations were calculated between each gene in the extended gene group and the average normalized gene expression of each cluster. We then also randomly selected one thousand genes from the no-evidence gene group, and calculated correlations between expression of these genes and the clusters. To obtain standard deviations, we performed this step one hundred times. The fraction of genes with a Pearson's correlation > 0.5 was then compared between both groups using Fisher's exact test (Figure 4C). We found that average gene expression in each cluster was more highly correlated with genes in the corresponding extended gene set than in the no-evidence gene set, particularly for Clusters A, B and G.

Based on an earlier report involving p53 targets [45], we decided to use the average normalized expression pattern of each cluster to predict new target genes for dominant transcription factors, First, we identified the genes with significant changes in gene expression in each gene group (*p *< 0.01). We then identified the subset of genes whose expression best matched each cluster using Pearson's correlations, and determined the relationship between the fraction of accepted genes (based on a range of cutoffs) that was contained in the extended gene set versus the "no evidence" gene set for each cluster (Figure 4D). As expected, all extended gene sets had higher accepted rates than the "no evidence" gene sets. However, as can be seen in Figure 4D, genes in the extended set for Clusters A and B were many times more likely to be matched the cluster aggregate expression profile than "no evidence" genes. This indicates that Cluster A and B expression profiles are better able to distinguish true member genes than the profiles for Cluster D or G. We identified 12 genes in the extended gene set for Cluster A and 24 for Cluster B that were highly correlated (*c *> 0.5) to the cluster aggregate expression profile (Table 3).

**Table 3:** Predicted genes for Cluster A and B from the extended gene sets.

Cluster	Gene Symbol	Gene Name
A	CFLAR	CASP8 and FADD-like apoptosis regulator
	CXCL1	chemokine (C-X-C motif) ligand 1
	EFNA1	ephrin-A1
	G0S2	G0/G1switch 2
	IL1R1	interleukin 1 receptor, type I
	IL1RN	interleukin 1 receptor antagonist
	OLR1	oxidized low density lipoprotein (lectin-like) receptor 1
	PLK3	polo-like kinase 3
	PTX3	pentraxin-related gene, rapidly induced by IL-1 beta
	RUNX1	runt-related transcription factor 1
	TNFAIP6	tumor necrosis factor, alpha-induced protein 6
	TNIP1	TNFAIP3 interacting protein 1

B	CASP4	caspase 4, apoptosis-related cysteine peptidase
	CD14	CD14 molecule
	CISH	cytokine inducible SH2-containing protein
	GBP1	guanylate binding protein 1, interferon-inducible, 67 kDa
	GBP2	guanylate binding protein 2, interferon-inducible
	GCH1	GTP cyclohydrolase 1
	HSPA1A	heat shock 70 kDa protein 1A
	IFI16	interferon, gamma-inducible protein 16
	IFIT3	interferon-induced protein with tetratricopeptide repeats 3
	IFITM1	interferon induced transmembrane protein 1
	IGF1R	insulin-like growth factor 1 receptor
	IL10RB	interleukin 10 receptor, beta
	IRF2	interferon regulatory factor 2
	ISG15	ISG15 ubiquitin-like modifier
	ISG20	interferon stimulated exonuclease gene 20 kDa
	JAK2	Janus kinase 2
	MX1	myxovirus (influenza virus) resistance 1, interferon-inducible protein p78
	PLSCR1	phospholipid scramblase 1
	RIPK1	receptor (TNFRSF)-interacting serine-threonine kinase 1
	SOCS1	suppressor of cytokine signaling 1
	STAT2	signal transducer and activator of transcription 2
	TIMP1	TIMP metallopeptidase inhibitor 1
	USP18	ubiquitin specific peptidase 18
	WARS	tryptophanyl-tRNA synthetase

Genes that showed high correlation to the average expression profile of a cluster (*c *> 0.5) were accepted as part of the cluster.

We also focused on Clusters A and B for predicting new target genes from the "no evidence" group. Some of the predicted new member genes for these clusters are listed in Table 4[46-48]. Although there was no evidence for including these genes in our model initially, we were able to partially validate certain target gene predictions based on evidence beyond the original knowledge-bases that we used to define our sets. Notable among this evidence was the use of genome-scale location analysis [46], as well as bioinformatics techniques [47] to detect NF-κB binding to the promoters of several predicted target sites. We conclude that such clustering may be useful for identifying new target genes, particularly in combination with other methods.

**Table 4:** Predicted genes for Cluster A and B form the "no evidence" group

Cluster	Category	Gene Symbol	Gene Name
A	Top 10	ETS2	v-ets erythroblastosis virus E26 oncogene homolog 2
		MTF1	metal-regulatory transcription factor 1
		SAMSN1	SAM domain, SH3 domain and nuclear localization signals 1
		IVNS1ABP	influenza virus NS1A binding protein
		IFNGR2	interferon gamma receptor 2
		PLAUR	plasminogen activator, urokinase receptor
		IL1R2	interleukin 1 receptor, type II
		AZIN1	antizyme inhibitor 1
		EHD1	EH-domain containing 1
		PCNX	pecanex homolog
	
	Related to innate immunity	IFNGR2	interferon gamma receptor 2
		IL1R2	interleukin 1 receptor, type II
		MAP4K4	mitogen-activated protein kinase kinase kinase kinase 4
		NCF1	neutrophil cytosolic factor 1
		TXN	thioredoxin
		RIPK2	receptor-interacting serine-threonine kinase 2
	
	Supported by other evidence	IFNGR2*	interferon gamma receptor 2
		GPR84**	G protein-coupled receptor 84
		FCAR**	Fc fragment of IgA, receptor for
		GADD45B**	growth arrest and DNA-damage-inducible, beta

B	Top 10	TRIM5	tripartite motif-containing 5
		GK	glycerol kinase
		SP110	SP110 nuclear body protein
		TMEM140	transmembrane protein 140
		CHMP5	chromatin modifying protein 5
		RHBDF2	rhomboid 5 homolog 2
		SAMD9	sterile alpha motif domain containing 9
		DDX58	DEAD (Asp-Glu-Ala-Asp) box polypeptide 58
		TLE3	transducin-like enhancer of split 3
		TRIM21	tripartite motif-containing 21
	
	Related to innate immunity	CSF2RB	colony stimulating factor 2 receptor, beta, low-affinity
		HCK	hemopoietic cell kinase
		IL13RA1	interleukin 13 receptor, alpha 1
		TLR1	toll-like receptor 1
		TNFRSF1A	tumor necrosis factor receptor superfamily, member 1A
	
	Supported by other evidence	DDX5***	DEAD (Asp-Glu-Ala-Asp) box polypeptide 5
		HERC5***	hect domain and RLD 5
		IFIT2***	interferon-induced protein with tetratricopeptide repeats 2
		OASL***	2'-5'-oligoadenylate synthetase-like
		SNX10***	sorting nexin 10
		ACSL4***	acyl-CoA synthetase long-chain family member 4

The ten most correlated target genes, as well as genes related to innate immunity (from the fifty most correlated genes) and genes which were supported by other evidence, are shown. *Predicted as a target gene from the motif sequence analysis. **DNA-protein binding detected in LPS stimulation. ***Up-regulated by a mutant of STAT1 which can activate expression in the absence of tyrosine phosphorylation.

### Overall regulatory dynamics in response to LPS

**Figure 5:**
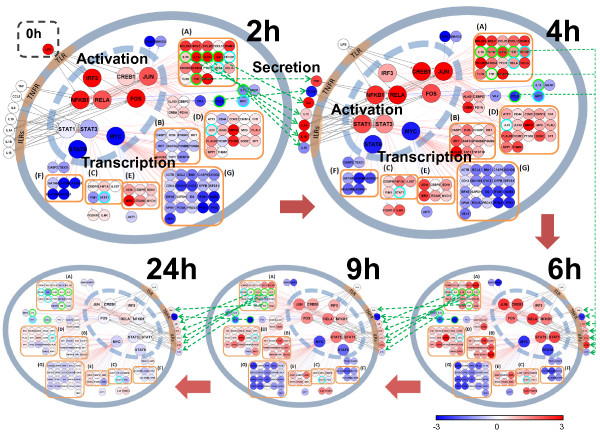
**A dynamic network of transcription**. At time zero, LPS is injected, giving rise to transcription factor activation, which then leads to induction or repression of gene expression, production and secretion of cytokines, and initiation of secondary signals. Target genes which correspond to secreted proteins (e.g., IL10, IL1A and IL1B) are noted with green circles, and transcription factors that are regulated by other factors, such as STAT1 and MYC, are noted with cyan circles. The seven major clusters marked in Figure 3A are grouped with orange boxes. Black lines denote activation of a transcription factor by an extracellular signal, red and blue lines show the influence of a transcription factor on a target gene, and green dotted lines indicate secretion of a gene product.

Finally, we were able to address our original goal of building an integrated temporal model of the human blood leukocyte response to LPS (Figure 5). This required the integration of our calculated transcription factor activities, transcription factor regulatory influences on each gene, clustering on the adjusted strength, and the gene expression data. Endotoxin was administered to the subjects at 0 hours. During the next two-hour period, IRF3, p65 and p50 were activated and interacted to regulate gene expression, as were JUN and FOS as well as CREB1. By two hours, these transcription factors had already affected gene expression, including the genes in Clusters A and D as well as the additional genes we predicted to belong in Cluster A. Between 2 and 4 hours after endotoxin administration, cytokines such as the interleukins (ILs) and tumor necrosis factor (TNF) whose genes were expressed at 2 hours were produced and secreted. These secreted proteins then and maintained or initiated the activity of several transcription factors in the blood leukocytes. Presumably, TNF then reactivated the NF-κB complex and some of ILs stimulated AP-1 complex [49,50]. In contrast, IRF3 activation rapidly returned the base level of activity. The ILs could have activated the STATs to initiate a secondary response, inducing expression of the genes in Clusters B and C together with the additional genes predicted to belong to Cluster B. After 4 hours, the transcription factors began to return to their basal level of activity, leading to a near-complete return to initial values of gene expression by 24 hours. The temporal model therefore provided a global view of activation, transcription and resolution of the blood leukocyte response to lipopolysaccharide in humans.

## Conclusion

The overall goal of this work was to build a dynamic network of transcription events following endotoxin administration from the time course response by global gene expression in peripheral blood leukocytes. From the expression profiles, we were able to predict the activities of ten transcription factors over time, as well as the regulatory strength a given transcription factor exerted on its target genes using NCA. Taken together, the activities often exhibited a high degree of correlation, both between factors and also between a factor's activity and its gene expression profile.

We also found that the regulatory strength matrix can be clustered to determine groups of genes which are not only co-expressed, but also co-regulated. Importantly, new and biologically relevant clusters were determined, suggesting that clustering by this approach is potentially more meaningful than methods which do not incorporate regulatory network information. Identification of these clusters also led us to identify many additional putative interactions between transcription factors and target genes not included in the known network, and most importantly, enabled us to describe and visualize the activation of regulatory proteins and target genes over time.

Certain limitations in both the available expression data as well as NCA itself could be addressed to make this approach more powerful. Gene expression analyses obtained from whole blood leukocyte samples provide an integrated signal from different leukocyte populations which are difficult to deconvolute, and so using a single cell population would be advantageous, such as could be obtained using cell sorting or other methods. Additionally, the number of transcription factors which can be used in NCA is approximately the number of expression profiles in the data set, and so a greater number of expression profiles – obtained at best shortly after the endotoxin administration – would also have been useful. Finally, NCA's scaling property, which makes it difficult to predict the direction of transcription factor activity, as well as NCA's current inability to incorporate time course information from the data set are important limitations to the method. Some approaches that may overcome these challenges include recent studies in which transcription factor activities were estimated using ordinary differential equation [45] or probabilistic models [51,52] of time course data. Future work might therefore focus on combining NCA with such efforts.

Notwithstanding these limitations, we were able to reconstruct the dynamics of endotoxin-dependent transcription in human peripheral blood leukocytes using the above results. This included identifying the activity of ten transcription factors regulating expression of ninety-nine genes. We also were able to identify additional genes that could be included in our model, notably 36 which had less initial evidence, but were substantiated by our predictions. Given that there were 1,215 genes with significant changes in gene expression for which regulatory relations were known, we were therefore able to capture between 8% (= 99 initial model genes/1,215 genes with significant expression changes and known regulatory relations) and 11% (99 + 36 additional genes = 135/1,215) of the explainable response. Furthermore, we were also able to identify new target genes based on the average gene expression profile of significant clusters, which could expand the scope of our temporal network still further. With a larger network reconstruction and data set specifically designed for use with NCA, it might be possible to move toward a near-complete characterization of dynamic transcription responses.

## Methods

### Data preprocessing and statistical analysis

To process our gene expression dataset prior to NCA, the log2 ratio of post-injection time points to the pre-injection time point was calculated. The significance of expression changes was then tested using one-way ANOVA, where the null hypothesis was that average gene expression levels were the same for each time point. We selected genes for our model if the ANOVA p-value was less than 0.01. Among 18,398 genes in the dataset, 5,518 genes were determined to be induced or repressed significantly. 1,215 of the genes that experienced a significant change in expression also had information about their regulation in the knowledge-bases we used.

### Network component analysis

NCA was developed by James Liao and colleagues [8]. Briefly, NCA models the expression of a gene as a linear combination of the activity of each transcription factor that controls the expression of the gene. Using this framework, NCA can estimate transcription factor activity and regulatory influence from a given regulatory network and a set of gene expression data. We followed the established method for generalized NCA, using a regularization factor of 0.8 to regulate the strength matrix *S *[13]. One important modification we made in our implementation of NCA was to normalize the transcription factor activity matrix. At each iteration step, A was normalized so that the norm of each row was 1. The S matrix was then also scaled as follows:



where *S*_•*j *_and *A*_*j*• _represent the *j*th column of *S *or row of *A*, respectively. This normalization stabilizes the calculation by preventing too large or too small values of *A*, but has no effect on the overall results due to NCA's scaling property [8].

### Hierarchical clustering on adjusted strength matrix

Having determined *S *using NCA, we wanted to use it for clustering genes. The first step was to enable comparison of transcription factor strengths to each other. A main challenge in such a comparison is that because of the scaling property of NCA [8], *S*_*ij *_and *A*_*jk *_are not unique solutions. However, the product *S*_*ij*_*A*_*jk *_is unique. Therefore, in order to enable clustering of the regulatory influences, we generated an adjusted strength matrix which is constant regardless of strength and activity. This matrix is calculated as follows:



where <,> represents the inner product of two vectors. We used hierarchical clustering to divide the adjusted strength matrix into meaningful clusters using angle cosine as the distance metric.

The dominant transcription factors associated with each cluster can be readily determined visually in this case. However, we also used a computational method to identify these dominant factors. This was accomplished by calculating a contribution factor for each transcription factor in a cluster. The contribution factor of transcription factor *j *for cluster *C *was calculated as the fraction of influence a given transcription factor imposed on the cluster with respect to the total influence of all *L *transcription factors, as follows:



Transcription factors for which the contribution factor was larger than 0.2 were chosen as dominant transcription factors of the cluster.

## Authors' contributions

JS designed the project, performed the analysis, and drafted the manuscript. WX provided the data, guided the analysis, and helped to draft the manuscript. LM revised the manuscript critically. RD conceived the study, and guided the research. MC supervised all aspects of the project. All authors read and approved the final manuscript.
